# Grain Refinement by Second Phase Particles under Applied Stress in ZK60 Mg Alloy with Y through Phase Field Simulation

**DOI:** 10.3390/ma11101903

**Published:** 2018-10-07

**Authors:** Yuhao Song, Mingtao Wang, Yaping Zong, Ri He, Jianfeng Jin

**Affiliations:** Key Laboratory for Anisotropy and Texture of Materials, Ministry of Education, School of Materials Science and Engineering, Northeastern University, Shenyang 110819, China; masteryhs@163.com (Y.S.); ypzong@mail.neu.edu.cn (Y.Z.); frankheri@stumail.neu.edu.cn (R.H.); jinjf@atm.neu.edu.cn (J.J.)

**Keywords:** phase field, ZK60 magnesium alloy, second-phase particles, recrystallization, grain growth

## Abstract

Based on the principle of grain refinement caused by the second-phase particles, a phase field model was built to describe the recrystallization process in the ZK60 alloy system with Y added under applied stress between temperatures 573 and 673 K for 140 min duration. The simulation of grain growth with second phase particles and applied stress during annealing process on industrial scale on the condition of real time-space was achieved. Quantitative analysis was carried out and some useful laws were revealed in ZK60 alloy system. The second phase particles had a promoting effect on the grain refinement, however the effect weakened significantly when the content exceeded 1.5%. Our simulation results reveal the existence of a critical range of second phase particle size of 0.3–0.4 μm, within which a microstructure of fine grains can be obtained. Applied stress increased the grain coarsening rate significantly when the stress was more than 135 MPa. The critical size of the second phase particles was 0.4–0.75 μm when the applied stress was 135 MPa. Finally, a microstructure with a grain size of 11.8–13.8 μm on average could be obtained when the second phase particles had a content of 1.5% and a size of 0.4–0.75 μm with an applied stress less than 135 Mpa after 30 min annealing at 573 K.

## 1. Introduction

ZK60 Mg alloy, widely used in many fields, has high strength compared with other commercial Mg alloys such as AZ31 [1]. However, ZK60 exhibits lower plasticity at room temperature, which limits its application as a structural material. One of the preferred methods to improve plasticity of the alloy is by the way of grain refinement, which in turn can be attained by pinning of second phase particles or quasi-crystal formed by adding certain elements (normally rear earth, e.g., Y) [2,3,4]. Research shows that microstructure evolution is affected by applied stress at elevated temperatures [5,6]. However, the effect of second phase particles on the grain size under applied stress has been investigated qualitatively or semi-quantitatively because of the difficulty and cost of precise control of second phase particles or quasi-crystal by experimental methods [3,4,7,8,9,10].

Thus, computer simulation methods to characterize the grain growth evolution under different experimental conditions are used, e.g., Monte Carlo [11,12], finite element [13] and phase field. Among them, phase field simulation method is effective in this area. Based on the thermodynamic and dynamic laws, phase field model can be employed to investigate the microstructure evolution process under conditions such as applied stress, electric field and so on [14,15,16,17].

Now, we can realize the simulation of microstructure evolution with second phase particles under applied stress by phase field method due to the efforts of researchers. There have been some attempts to study the microstructure evolution in the existing of second phase particles and applied stress separately. For instance, Moelans et al. modified the free energy expression by introducing a new field variable to describe inert and incoherent second phase particles [18]. Kunok et al. evaluated the effect of shape, distribution and size of second phase particles on the microstructure in 2D and 3D [19,20,21,22]. He et al. successfully realized the simulation of microstructure evolution in a real alloy system under the real temporal and spatial conditions on industrial scale [23]. As to applied stress or strain effect, the elastic energy caused by applied stress or stress can be coupled into phase field model. Wen et al. examined the influence of an applied homogeneous strain on the transformation of coherent α_2_ to *O*-phase in Ti–Al–Nb system [24]. Guo et al. employed the phase-field model coupled with elastic energy to investigate the effect of a superimposed stress on the Ni_4_Ti_3_ particle coarsening process [25].

In this study, first, a phase field model including both elastic energy and the second phase item was established. Real values of the model parameters in ZK60 alloy system were determined according to a set of rules proposed by Wang et al. [26], which led to results consistent with real microstructure evolution process. Thus, the effect of second phase particles and applied stress on the microstructure characteristics could be quantitatively evaluated. Then, we used the model to investigate the influence of second phase particles and applied stress on grain growth of ZK60 Mg alloy with the addition of Y, under real temporal and spatial conditions that are compliant with industrial scale. Finally, a proper prediction was made for the alloy design and process optimization in our target alloy system.

## 2. Model and Parameters

### 2.1. Phase Model with Second Phase Particles and Elastic Energy

With the rapid development of phase field method, it is available for the simulation of a system with second phase particles and applied stress or stain [15,18]. However, there is no single model containing the two influence factors together to date. In our study, a phase field model coupling both the second phase particles and applied stress was developed. Here, with the value determination rules proposed by our team [26], the microstructure evolution of ZK60 Mg alloy with the addition of Y is discussed.

According to the features of target alloy microstructure, a set of variables relating to orientation and concentration were chosen to describe the grains with different orientations. All variables can be obtained by solving the time-dependent Ginzburg–Laudon equation [27] and Cahn–Hill diffusion equation [28] as follows,

(1) $$\frac{\partial \eta_{p}\left(r,t\right)}{\partial t}=-L\frac{\delta F}{\delta \eta_{p}\left(r,t\right)},\;p=1,2,3\ldots n$$

(2) $$\frac{\partial c\left(r,t\right)}{\partial t}=M\nabla^{2}\frac{\delta F}{\delta c\left(r,t\right)}$$

where *η* is long-range order parameters, *t* is time, *r* is position factor, *p* is orientation, *c* is concentration of Zn, *L* is a variable related to grain coarsen mobility, *M* is chemical mobility and *F* is the function of free energy and can be expressed as follows in our system.

(3) $$F=F_{ch}+E_{el}$$

where $F_{ch}$ is chemical free energy and $E_{el}$ is elastic strain energy. $F_{ch}$ can be expressed as follows

(4) $$F_{ch}=\int \left[f_{0}\left(c,\eta_{1}\left(r,t\right),\eta_{2}\left(r,t\right),\ldots ,\eta_{n}\left(r,t\right)\right)+\frac{K_{2}}{2}\overset{n}{\underset{q=1}{\sum }}{\left(\nabla \eta_{q}\left(r,t\right)\right)}^{2}\right]dr$$

(5) $$f_{0}=A+\frac{A_{1}}{2}{\left(c\left(r,t\right)-c_{l}\right)}^{2}+\frac{A_{2}}{4}{\left(c\left(r,t\right)-c_{l}\right)}^{4}-\frac{B_{2}}{2}{\left(c\left(r,t\right)-c_{l}\right)}^{2}\overset{n}{\underset{q=1}{\sum }}\eta_{q}^{2}\left(r,t\right)+\frac{B_{2}}{4}\overset{n}{\underset{q=1}{\sum }}\eta_{q}^{4}\left(r,t\right)\;+\frac{K_{2}}{2}\overset{n}{\underset{q=1}{\sum }}\overset{n}{\underset{p\neq q}{\sum }}\eta_{q}^{2}\left(r,t\right)\eta_{p}^{2}\left(r,t\right)+\varepsilon \phi \left(r\right)\overset{n}{\underset{q=1}{\sum }}\eta_{q}^{2}\left(r,t\right)$$

In Equation (5) [23], f_0_ is local specific free energy relating to thermodynamic properties of the system. *A*, *A*_1_, *A*_2_ and *B*_2_ are coefficients related to free energy. *K*1 is coupling item coefficient between two adjacent grains with different orientations, represented by *p* and *q*, *K*_2_ is related with grain boundary features. ε is a parameter with the pinning ability of second phase particles.

To study the effect on the microstructure evolution caused by applies stress, the elastic energy item based on elastic theory proposed by Chen [29] is employed in our model and the expression is as follows.

(6) $$E_{el}=\frac{V}{2}C_{ijkl}{\bar{\varepsilon }}_{ij}{\bar{\varepsilon }}_{kl}-VC_{ijkl}{\bar{\varepsilon }}_{ij}\underset{p}{\sum }\varepsilon_{kl}^{0}\left(p\right)\bar{\eta_{p}^{2}\left(r,t\right)}$$

where *V* is volume and *C_ijkl_* is elastic tensor for polycrystallization system ${\bar{\varepsilon }}_{ij}$ and $\varepsilon_{kl}^{0}$ means eigenstrain whose value depends on each grain orientation. To connect the grain orientations and applied stress, each *η* stands for a special orientation by giving a special Euler angle [29].

Above all, our phase field model is based on orientation variables (non-conserved) and composition variables (conserved), including a function of free energy. The free energy function is essential to a phase model, including chemical energy and elastic energy in our alloy system. Finally, the whole model is set up by coupling items of second phase particles and elastic strain energy with polycrystal grain growth model.

### 2.2. Model Parameters Setting and Initial Condition

The alloy chemical composition is *ω* (Zn) = 4.9%, *ω* (Zr) = 0.7%, *ω* (Y) = 0% (for parameters calibration and model test) and 0.9% with rest of Mg. According to Reference [2], the alloy production process includes melting at 993 K, ingot casting, hot rolling at 673 K with a reduction rate of 95% and annealing at 573–673 K. In our research, the annealing process was simulated and the temperatures are 573 K, 623 K and 673 K.

To accomplish the simulation under the real temporal and spatial condition, it is essential to determine the real physical values of all the parameters in our model. The local free energy is a function of composition and phase, the parameter *η* represents phase and, thus, the free energy of the system can be expressed with composition *c* and *η*. The concrete expression is shown in Equation (5). Considering the limited computing resource, we implement the simulation in a 2D system with 36 orientations whose corresponding Euler angles are determined by the same way in Reference [30]. *ε* is the coefficient of second phase particles item and its value shows the pinning effect on the grain boundary by the particles. We take *ε* as 1.0 in our model. Moelans [18] introduced ϕ(r) into local free energy function *f*_0_ to simulate the grain growth behavior in an alloy system with second phase particles which is independent with time. According to Equation (5), the grid point in position *r* is inside a second phase particle when ϕ(r)=1, and the item $\varepsilon \phi \left(r\right)\sum_{q=1}^{n}\eta_{q}^{2}\left(r,t\right)$ is non-zero so that it contributes to *f*_0_. Otherwise, a grid point is outside a particle when ϕ(r)=0, and the item $\varepsilon \phi \left(r\right)\sum_{q=1}^{n}\eta_{q}^{2}\left(r,t\right)$ does not serve any function because it equals zero.

The derivation of local free energy against *η* has to be zero when *η* = 0 to keep *f*_0_ minimum at *η* = 1. That means the relation between *B*_1_ and *B*_2_ must satisfy the following,

(7) $$B_{1}{\left[c\left(r,t\right)-c_{l}\right]}^{2}=B_{2}$$

In Equation (5), the microstructure before and after recrystallization can be represented by *f*_0_ with different *η*, namely, $\eta_{p}^{2}=1$ and $\sum_{q\neq p}^{n}\eta_{q}^{2}=0$ means the state after recrystallization and $\sum_{q=1}^{n}\eta_{q}^{2}=0$ represents that before recrystallization. Thus, the released stored energy caused by deformation can be derived as follows,

(8) $$E=f_{0}\left(\overset{n}{\underset{q=1}{\sum }}\eta_{q}^{2}=0\right)-f_{0}\left(\eta_{p}^{2}=1,\overset{n}{\underset{q\neq p}{\sum }}\eta_{q}^{2}=0\right)=\frac{B_{1}}{2}{\left(c-c_{l}\right)}^{2}-\frac{B_{2}}{4}$$

It is reported that the stored energy will increase with the increase of pre-strain and approach a constant when the pre-strain is over 0.2 [31]. It is taken as 0.54 J g^−1^ namely, *E* = 12.8 J mol^−1^, because the pre-strain of the alloy is 0.95 in Reference [2]. The values of *B*_1_ and *B*_2_ can be obtained as *E* = 12.8 J mol^−1^ by solving Equations (7) and (8). *B*_1_ and *B*_2_ at different temperatures are shown in Table 1.

**Figure 1:**
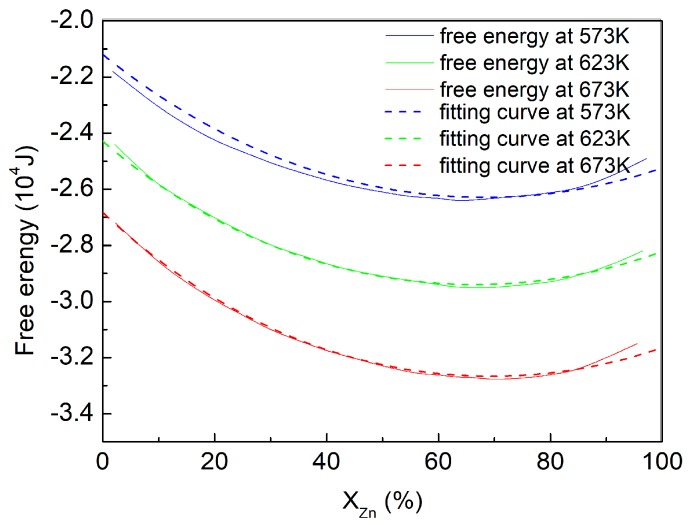
The free energy-component curve of ZK60 Mg alloy obtained by the software Thermo-Calc at 573 K, 623 K and 673 K, and their fitting curves.

We use Thermo-Calc thermodynamic software to calculate the free energy–concentration curve of our target alloy at different temperatures (Figure 1). The parameters *A*, *A*_1_ and *A*_2_ are obtained by fitting Equation (5) with $f_{0}\left(\eta_{p}^{2}=1,\sum_{q\neq p}^{n}\eta_{q}^{2}=0\right)$. The values of *A*, *A*_1_ and *A*_2_ are obtained from fitting curves, which is also shown in Figure 1.

The grain boundary scope in ZK60 Mg alloy should be between 1.0 μm and 2.0 μm and is mainly decided only by *K_2_* [26]. Then, if the grain boundary range is taken as 1.172 μm (8 grids), *K*_2_ can be determined as 4.4 × 10^−12^ J∙m^2^/mol. Both *K*_1_ and *K*_2_ influence the grain boundary energy, therefore we can determine *K*_1_ by the grain boundary energy calculation formula with the value of *K*_2_ and the grain boundary energy. Normally, the random large angle grain boundary ranges from 0.5 to 0.6 J/m^2^ in polycrystalline microstructure and 0.55 J/m^2^ was chosen in our research [32]. *K*_1_ is calculated as 2.05 × 10^2^ J/mol in this way (Table 1).

The elastic constants of the ZK60 alloy in the simulation are C_11_ = 62.3 GPa, C_12_ = 25.5 GPa, C_13_ = 23.1 GPa, C_33_ = 66.2 GPa, C_44_ = 14.1 GPa, and C_66_ = 18.4 GPa [32].

**Figure 2:**
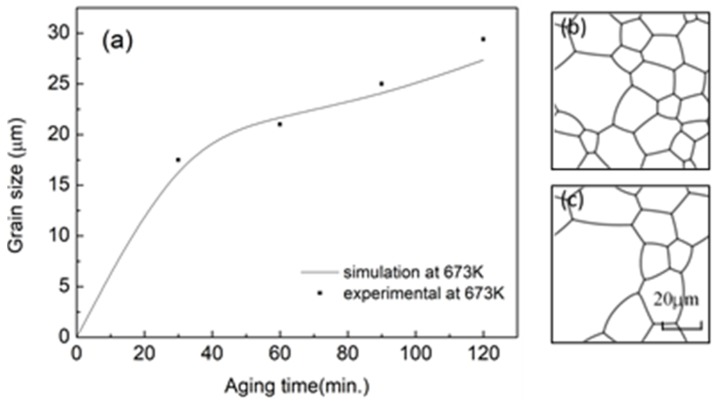
(**a**) The comparison of grain size; (**b**,**c**) simulation microstructure at 30 min and 120 min, respectively.

*M* in Equation (2) is taken as chemical mobility of Zn in target alloy in our model, whose value is 3.87 × 10^−12^ m^2^∙mol/(J∙s) [33]. According to Reference [26], *L* in Equation (1), related to the grain boundary mobility, is a function of temperatures and can be expressed as follows,

(9) $$L=L_{0}e^{-\frac{Q}{RT}}$$

where *R* is the gas constant, *Q* is segregation activity energy of Zn in our simulation and *L*_0_ is a constant depending on target alloy system. However, *L*_0_ cannot be derived directly, and we calibrated it by comparison of simulation and experimental data at some temperature, which was chosen as 673 K in our simulation [2]. It was found that the grain size simulation curve fitted (see Figure 2) the experimental results well when *L* = 51.41 × 10^−3^ mol/(J∙s) at 673 K. Thus, *L_0_* was found to be 7.23 mol/(J∙s). Then, the *L* at 573 K and 623 K, which is a common heat treatment temperature of alloy with the second-phase, can be calculated to be 21.7 × 10^−3^ mol/(J∙s) and 34.56 × 10^−3^ mol/(J∙s), respectively. The simulation results were compared with experimental data, as shown in Figure 2 and both the grain size and the microstructure morphology match well with those obtained from experiments [2].

Above all, a phase field model applied to the microstructure evolution with the second phase particles in ZK60 Mg alloy under applied stress was built. All parameters have a clear physical meaning and were confirmed uniquely through theoretical calculation and experimental data calibration. Utilizing this phase field model, the influence of the second phase particles and applied stress on micro characteristics can be studied quantitatively and the simulation results are real on industrial scale.

The process of nucleation was simulated by a phenomenological method. The initial nucleation state was distributed randomly on the grain boundary, and the radius of each nucleus was set as seven grids. The simulation was run on the 768 × 768 2D uniform grids, and the size of each cell is 0.1465 μm × 0.1465 μm. The boundary condition of the phase field was defined as the periodic boundary.

## 3. Result and Analysis

### 3.1. Recrystallization

Normally, the annealing temperature for ZK60 deformed Mg alloy is between 573 K and 673 K [2]. In this study, the recrystallization process of the system without second phase particles and applied stress was simulated at 573 K and 623 K and a comparison between experimental data and simulations was made for our model testing.

**Figure 3:**
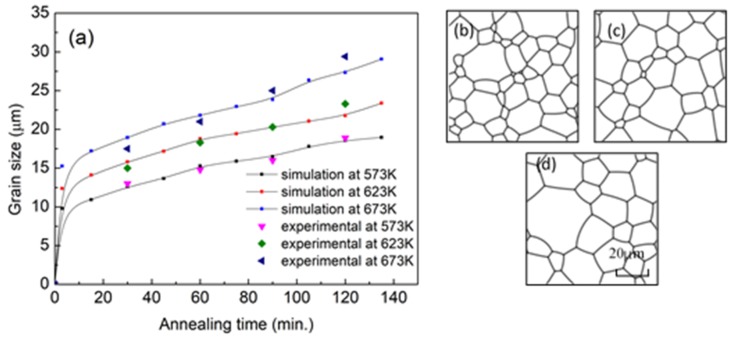
(**a**) The comparison of grain size; (**b**) simulation microstructures at 573 K and 30 min; (**c**) simulation microstructures at 623 K and 30 min, (**d**) simulation microstructures at 673 K and 30 min.

The comparison between the experimental results and simulations is shown in Figure 3a. The curve at 673 K was chosen to calculate model parameters and the simulation data were compared with experimental results [2] (Figure 2). Therefore, both the grain size and the microstructure morphology match well with those obtained from experiments. At 573 K and 623 K, the experimental data also match well with the simulations (Figure 3a). Meanwhile, the microstructure morphology obtained by experiments in reference [2] also resembles that our simulation results. Through comparison, the maximum difference of average grain size between the experimental and simulation data happens at 623 K and 120 min and the difference is 1.5 μm, i.e. less than 7%.

Based on the comparison of grain size and morphology, we can say that there is good agreement between the experimental results and simulations, which indicates the correctness and validation of this model and parameters in our target alloy.

### 3.2. Recrystallization with Second Phase Particles

Second phase particles, i.e. Mg_3_Zn_6_Y after Y is added into ZK60 alloy, form at elevated temperatures and will be broken into small pieces during hot rolling process [2]. The second phase particles diffusely distribute in the alloy and have a strong pinning effect on grain boundary during recrystallization. Referring to the shape and size of Mg_3_Zn_6_Y in experimental observation, the second phase particles, having four directions and a size of 0.3 μm (in length), was added in our model.

**Figure 4:**
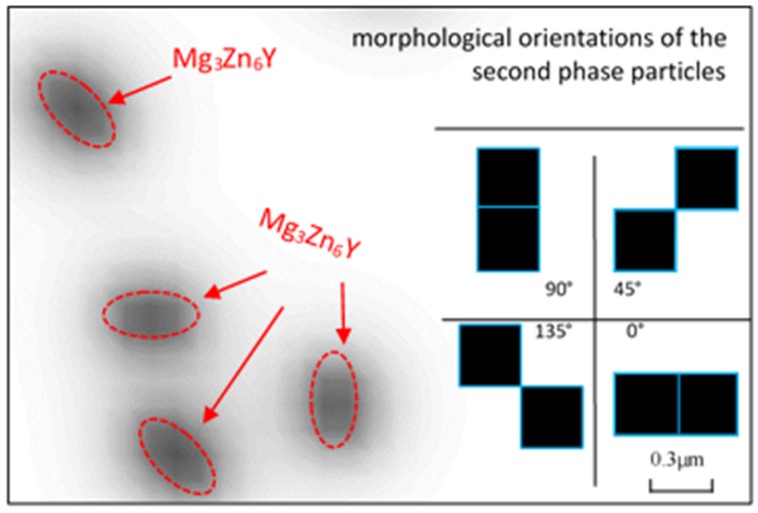
Second phase particles setting in phase field model.

The second phase particles in our model are presented in Figure 4. The particles are distributed inside and outside grain boundary area randomly in our model. The morphological orientations of Mg_3_Zn_6_Y are just like that in the reference [2].

**Figure 5:**
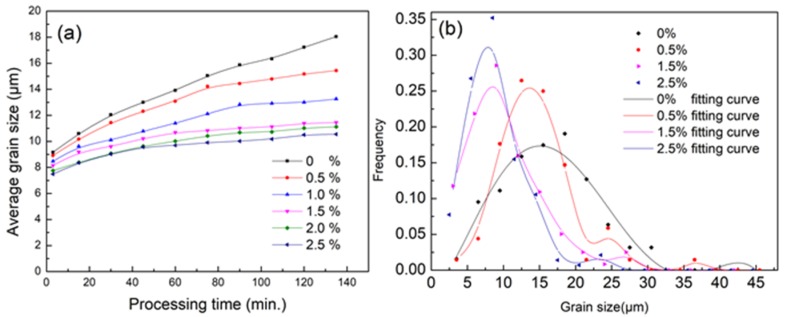
(**a**) The average grain size evolution at 573 K with second phase particles of different contents; and (**b**) the distribution of grain size annealing at 60 min.

Second phase particles have a hindering effect on the movement of grain boundaries during recrystallization, and the particle content plays an important role on the grain size. In this study, a qualitative analysis with different Mg_3_Zn_6_Y contents by phase field simulation was carried out, and the results are shown in Figure 5. At elevated temperatures, the whole system energy will decrease through grain boundary movement. With the existence of second phase particles, the boundary movement will be hindered and the average grain growth will be inhibited as well. More particles mean leads to boundary movement resistance and smaller average gran size, which is consistent with Figure 5a. With the increasing particle content (0–2.5%), the average grain size decreases significantly. However, the hindrance caused by second phase particles is not increasing linearly with the content. According to Figure 5a, the grain coarsening rate decreases from 0.058 to 0.019 μm/min and the rate becomes stable when the content exceeds 1.5%, which means a significant weakening effect in ZK60 Mg alloy.

**Figure 6:**
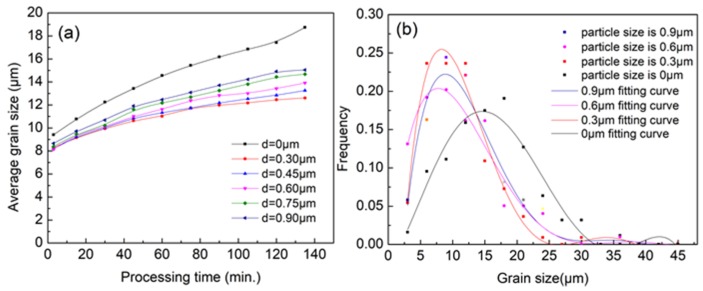
(**a**) The average grain size evolution at 573 K with second phase particles of different size; and (**b**) the distribution of grain size with processing 60 min.

After the discussion of the effect on grain boundary movement by the particle content, a further computation on the influence of the particle size was carried out. It was proven that larger particles have a stronger binding effect on the grain boundary by experimental observations. However, it is difficult to change both particle size and number under the condition of a fixed particle content in experiments. Thus, the above rule is mainly verified on the premise that both the size and content of particles increase together. Under the condition of fixed particle content, the size and number of particles are negatively correlated, namely, larger particles mean fewer particles, and vice versa. Phase field model can easily study the microstructure evolution under the above condition, which is difficult and costly by means of experiments. According to the results above, the particle content of 1.5% was chosen as the initial condition, and the results are shown in Figure 6a. With a fixed particle content of 1.5%, the refinement effect of second phase particles on grain size increases at first and then decreases when the size of particles increases from 0 to 0.9 μm.

There is a critical value of 0.3 μm in the size of particles, which has a best refinement effect on the grain size. It is also found that the peak value of grain size decreases first and then increases with the increase particle size (see Figure 6b). The main reason for this phenomenon is that, when the size of the second phase particle is small, there will be little pinning effect on the grain boundary. Therefore, even a large number of particles will have no effect on grain refinement. Besides, the pinning effect gradually increases with an increasing particle size. When the pinning effect caused by the increase of particle size becomes weaker than that caused by the decrease of particle number, the further increase of particle size will weaken the effect of grain refinement, namely, the refinement effect is reduced. We can imagine an ultimate state, in which the particle size is big enough that the particle number reduces to one, then the particle will only affect the surrounding grains and there is no pinning effect on the grains in other area so that the refinement effect will be also very weak in this condition. The relationship between the second phase particle size and average grain size is shown in Figure 6a.

### 3.3. Applied Stress

**Figure 7:**
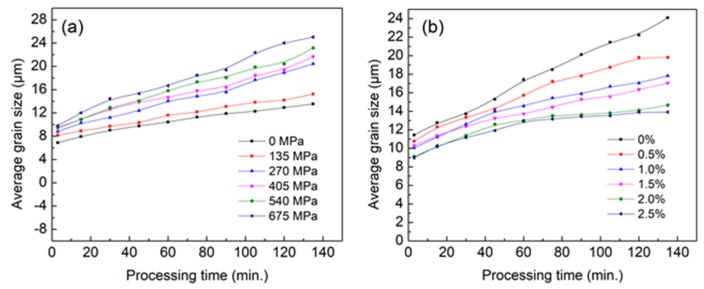
(**a**) Average grain size under different stress at 573 K; and (**b**) the average grain size evolution at 573 K with second phase particles of different content.

During the deformation of polycrystalline materials, the growth rate of grains having different orientations is different due to the influence of applied stress. We used the phase field model coupling elastic energy to study the effect of applied stress on the evolution process of microstructure of target alloys. The stress conditions and results are shown in Figure 7a. Simulation shows that applied stress not only affects the average grain size but also increases the coarsening rate of grains, as shown in Figure 7a. At 573 K, when the external stress exceeded 135 MPa, the coursing rate increases significantly, from 0.06 to 0.09 (see Figure 7a). The main reason for this phenomenon is that the driving force for the growth of grains with different orientations is not only grain boundary curvature, but also the elastic energy caused by external stress under the conditions of applied stress. The whole system evolves with the reduction of interface energy and elastic energy. Therefore, the grains with the orientations with lower elastic energy can get a greater advantage in growth. That means elastic energy exits as a kind of driving force for grain growth. If there is a considerable difference of elastic energy caused by orientations to applied stress between adjacent grains, grains with preferential orientation growth speed will increase, and a larger applied stress means greater elastic energy difference, and also leads a greater average grain growth speed.

Finally, we used our model to study the evolution characteristics of the microstructure in the presence of both applied stress and second phase particles. In this study, the applied stress was taken as 135 MPa considering that the grain coarsen becomes more obvious when the applied stress exceeds 135 MPa. The size and content of second phase particles were 0.3 μm and 0–2.5%, respectively. The results are in Figure 7b which also shows the pining effect and a weakening effect with a content exceeding 1.5%.

**Figure 8:**
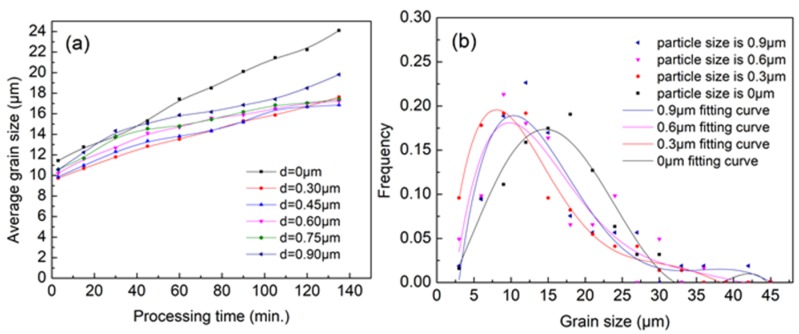
(**a**) The average grain size evolution at 573 K with second phase particles of different size under 135 MPa stress; and (**b**) the distribution of grain size with processing 60 min.

Based on the above, we studied the influence rule of particle size on microstructure under the condition of 1.5% content and external stress of 135 MPa. As shown in Figure 8a,b, the rule is basically consistent with the previous conclusion. However, the size of the critical particles increases from 0.3 μm to 0.4–0.7 μm. This is because, when the external stress field exists, the existence of elastic energy enhances the driving force of the dominant grains, which requires larger particles to provide greater resistance. Therefore, the size of the critical particles also increases.

**Figure 9:**
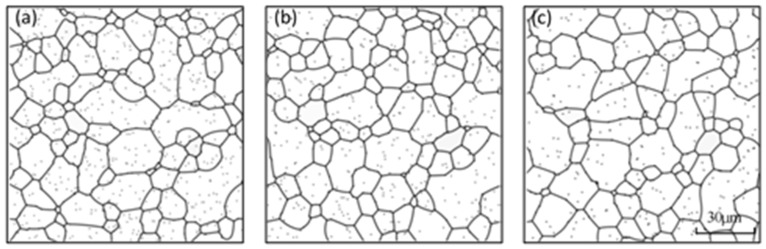
The simulation microstructure at 573 K after annealing for 30 min under a 135 MPa applied stress with a particle size: (**a**) 0.3 μm; (**b**) 0.45 μm; and (**c**) 0.6 μm.

Above all, the microstructure evolution of ZK60 Mg alloy with Y under the applied stress at elevated temperature was simulated on industrial scale on the condition of real time-space by a phase field method. We used a phase field model coupling elastic energy and the second phase items to discuss an effective way of grain refinement in the alloy. Quantitative research was carried out to study the microstructure characters affected by the content and size of second phase particles with and without applied stress. According to our simulation results, when the second phase particles has a content of 1.5% and a size of 0.4–0.75 μm with an applied stress less than 135 MPa after 30 min at 573 K, a microstructure with an average grain size of 11.8–13.8 μm can be obtained (Figure 9). We discuss the effect on the grain orientation of the second phase particles and applied stress in our following paper.

## 4. Conclusions

1. The grain growth with the second phase particles and applied stress existing during annealing process was simulated on industrial scale on the condition of real time-space by a phase field method.
2. The second phase particles have a promoting effect on the grain refinement; however, the effect weakened significantly when the content exceeded 1.5%. When the content is 1.5%, there exists a critical range of second phase particle size 0.3–0.45 μm, within which a microstructure of fine grains can be obtained.
3. Applied stress can increase the grain coarsening rate significantly when the stress is more than 135 MPa. Moreover, second phase particles also have a significant refinement effect with the existence of applied stress. As to the critical size of the second phase particles, a critical range for the second phase particles, 0.4–0.75 μm, still exits under an applied stress of 135 MPa.
4. A microstructure with a grain size of 11.8–13.8 μm on average can be obtained when the second phase particles have a content of 1.5% and a size of 0.4–0.75 μm with an applied stress less than 135 MPa after 30 min annealing at 573 K.

## Figures and Tables

**Table 1:** Values of the parameters in the function of local free energy.

Temperature (K)	*A* (kJ/mol)	*A*_1_ (kJ/mol)	*A*_2_ (kJ/mol)	*B*_1_ (J/mol)	*B*_2_ (J/mol)	*K*_1_ × 10^2^ (J/mol)	*K*_2_ × 10^−12^ (J∙m^2^/mol)
573	−26.3	20.1	8.3	138.05	51.2	2.05	4.4
623	−29.4	20.2	12.5	128.82	51.2	2.05	4.4
673	−32.7	21.2	11.9	121.93	51.2	2.05	4.4
